# Reduced strigolactone exudation as a key resistance mechanism in wild carrots against *Phelipanche aegyptiaca*

**DOI:** 10.1093/pcp/pcaf113

**Published:** 2025-09-18

**Authors:** Sukhmanpreet Kaur, Mwafaq Ibdah, Riko Sakioka, Kyogo Nagano, Kaori Yoneyama, Philipp Simon, James Westwood, Dorothea Tholl

**Affiliations:** Department of Biological Sciences, 409 Latham Hall, 220 Ag-Quad Lane, Virginia Tech, Blacksburg, VA 24061, USA; School of Plant and Environmental Sciences, 401 Latham Hall, 220 Ag-Quad Lane, Virginia Tech, Blacksburg, VA 24061, USA; Plant Sciences Institute, Newe Ya’ar Research Center, Agricultural Research Organization (ARO), P.O. Box 1021, Ramat Yishay 30095, Israel; Research and Development Bureau, Saitama University, Saitama 338-8570, Japan; Research and Development Bureau, Saitama University, Saitama 338-8570, Japan; Research and Development Bureau, Saitama University, Saitama 338-8570, Japan; Vegetable Crops Research Unit, United States Department of Agriculture, 1575 Linden Drive, Madison, WI 53706-1514, USA; School of Plant and Environmental Sciences, 401 Latham Hall, 220 Ag-Quad Lane, Virginia Tech, Blacksburg, VA 24061, USA; Department of Biological Sciences, 409 Latham Hall, 220 Ag-Quad Lane, Virginia Tech, Blacksburg, VA 24061, USA

**Keywords:** carrot, Egyptian broomrape, Phelipanche aegyptiaca, *P. aegyptiaca* germination, *P. aegyptiaca* tubercles, post-attachment resistance, pre-attachment resistance, root exudation, strigolactones, wild carrots

## Abstract

*Phelipanche aegyptiaca* is a root parasitic plant that causes significant yield losses in many crops, including carrots (*Daucus carota*). This study investigates the resistance mechanisms of two wild carrot accessions, PI 21793 (*Daucus glaber*) and PI 341902 (*Daucus littoralis*), against *P. aegyptiaca* compared to a cultivated carrot (P0114; *D. carota*). Wild carrots induced lower germination rates of *P. aegyptiaca* seeds and fewer successful tubercles, indicating both pre-attachment and partial post-attachment resistance mechanisms. Strigolactone analysis revealed significant quantitative differences between cultivated and wild carrots. While cultivated carrots exuded high levels of two strigolactones, one of which was putatively identified as the non-canonical strigolactone, 4-oxo-methyl-carlalactone, wild carrots released lower amounts of these compounds. Supplementation with the artificial strigolactone analog GR24 increased germination in *P. aegyptiaca* inoculated on wild carrots, suggesting that strigolactone deficiency and possibly altered composition are key pre-attachment resistance mechanisms. However, higher germination resulted in no significant improvement in tubercle development on wild carrots. Parasite seedlings showed necrosis-like symptoms at their attachment sites on wild carrot roots, indicating an additional post-attachment resistance mechanism. These findings provide new insights into strigolactone-mediated host resistance and highlight the potential of wild carrot accessions to contribute to the development of resistant cultivars against parasitic plants.

## Introduction

*Phelipanche aegyptiaca* (Egyptian broomrape) is a parasitic plant that thrives by invading the roots of a wide variety of plants from several families such as the Solanaceae, Brassicaceae, Fabaceae, and Apiaceae (Cochavi et al. 2016, Iasur Kruh et al. 2017, Nativ et al. 2017). It is particularly prevalent in the Mediterranean region, including in Israel, where it has caused up to 100% yield losses in many crops from families of the Solanaceae, Fabaceae, Compositae, Cruciferae, and Umbelliferae resulting in an economic loss of around $15 million per year (Cohen et al. 2017, Aly et al. 2019). A significant challenge in controlling *P. aegyptiaca* is its late emergence, as it may grow for weeks in the soil hidden from view, rendering it largely undetectable to farmers. *P. aegyptiaca* seeds can remain dormant in the soil for several years until optimal environmental conditions and the presence of a suitable host plant trigger their germination (Joel and Bar 2013). The conditioning phase required for seed germination involves several days of warm weather and moisture (Joel et al. 2012), after which the seeds of *Phelipanche* species exhibit the capacity to germinate upon host-derived chemical signals such as strigolactones (Fernandez-Aparicio et al. 2016). Following germination, the emerging germ tubes of the parasite may grow up to a few millimeters to establish contact with host roots. The parasite subsequently forms a specialized organ, the haustorium, which invades the host root and develops vascular connections to absorb nutrients and water from the host (Joel et al. 2012). In the subsequent stage, *P. aegyptiaca* tissues outside the host root expand to form a tubercle that acts as a storage organ (Ekawa and Aoki 2017). This tubercle then initiates a shoot that emerges from the soil and bears flowers for reproduction. Notably, the emergence of this aerial shoot represents the only visible stage of the parasite aboveground (Nativ et al. 2017), yet significant crop damage can occur even prior to the appearance of *P. aegyptiaca* shoots (Cohen et al. 2017).

Carrot (*Daucus carota*), which is an agriculturally and economically important crop worldwide (Ahmad et al. 2019), is heavily parasitized by *P. aegyptiaca* in many countries including Israel (Emran et al. 2020). Parasitism of carrot leads to low sugar content and decreased taproot biomass, resulting in a decline in carrot quality and quantity (Cochavi et al. 2016). Control of *P. aegyptiaca* in carrot has been achieved with sequential applications of glyphosate (Cochavi et al. 2015), but these applications are labor-intensive, time-consuming, and expensive, while also potentially detrimental to the crop. Therefore, identifying resistance mechanisms in crops or crop relatives is crucial for developing more effective and sustainable control strategies (Aly 2012). Limited research has been conducted on the resistance of carrots against root parasites. To date, only a single study has been published, documenting post-attachment resistance in two French carrot varieties (*D. carota*) against *Phelipanche* (syn. *Orobanche*) *ramosa,* a root parasite closely related to *P. aegyptiaca* (Zehhar et al. 2003)*.*

Host resistance to root parasitic plants is broadly divided into two categories: pre- and post-attachment resistance. Pre-attachment mechanisms generally involve the chemical signaling that triggers parasite seed germination, while post-attachment mechanisms include processes that prevent haustorial penetration or subsequent feeding by the parasite. Investigations into other host plants and similar parasitic plant species have unveiled resistance mechanisms preceding parasite attachment (Albanova et al. 2023). Strigolactones play a pivotal role in pre-attachment resistance mechanisms (Jhu and Sinha 2022). These plant hormones serve dual purposes: internally, they regulate shoot branching, and externally, they facilitate interactions with arbuscular mycorrhizal fungi (Smith 2014). Parasitic plants exploit these external signals to their advantage to initiate their germination (Yoneyama et al. 2010). Host plant resistance can involve alteration of strigolactone exudation or composition, which can impede parasite germination and attachment (Albanova et al. 2023). For example, maize genotypes that predominantly exude the strigolactone, zealactol, exhibit reduced damage from the root parasite *Striga* compared to those producing the major maize strigolactone, zealactone (Li et al. 2023). Similarly, a maize variety releasing 5-deoxystrigol demonstrated increased susceptibility to *Striga*, whereas a variety exuding sorgomol exhibited resistance (Yoneyama et al. 2015). Furthermore, strigolactone deficiency and the reduced release of strigolactones observed in a tomato line and pea genotypes, respectively, resulted in resistance against root parasites (Dor et al. 2011, Pavan et al. 2016). Moreover, a mutation study demonstrated that tomato mutants lacking core gene functions in the strigolactone biosynthetic pathway (CCD7 and CCD8) led to complete resistance against *P. aegyptiaca* (Karniel et al. 2024).

In contrast to pre-attachment resistance, post-attachment resistance mechanisms occur following parasitic plant haustorium formation and function by impeding parasite development within the host plant. For instance, host plants utilize various defensive strategies such as releasing cytotoxic compounds like phenolic acids and coumarins or inducing lignification of cell walls to hinder parasite penetration (Faradonbeh et al. 2020, Albert et al. 2021). Additionally, an increase in reactive oxygen species activity can further inhibit or delay parasite development (Albert et al. 2021). Importantly, even in the case of successful broomrape attachments, certain host genotypes from sesame (*Sesamum indicum*), clover (*Trifolium* spp.), and vetch (*Vicia sativa* L.) can effectively prevent parasite reproductive development (shoot formation) by causing necrosis of parasite tubercles (Ross et al. 2004, Teimouri Jervekani et al. 2016).

Historically, breeding programs prioritizing yield enhancement have inadvertently reduced genetic diversity, compromising biotic and abiotic stress tolerance in crops (Rispail et al. 2007). Therefore, wild crop relatives offer a promising avenue to uncover novel resistance mechanisms against parasitic plants as has been observed in a wild maize relative (*Tripsacum dactyloides*) against *Striga hermonthica* and wild vetch (*Vicia* spp.) against *Orobanche crenata* (Rispail et al. 2007). In a similar approach, the present study investigated *P. aegyptiaca* resistance of a wild carrot (*D. glaber*) accession collected in Israel. Specifically, we aimed to identify the basis for differences in parasite resistance between wild and cultivated carrot accessions. To achieve this, we tracked *P. aegyptiaca* growth from germination to tubercle formation and analyzed root exudates using LC–MS/MS. Here, we demonstrate that strigolactone levels explain a major component of the resistance in wild carrot, but there is also evidence for a post-attachment resistance mechanism.

## Results

### Wild carrots are less parasitized by *P. aegyptiaca* than cultivated carrots

**Figure 1:**
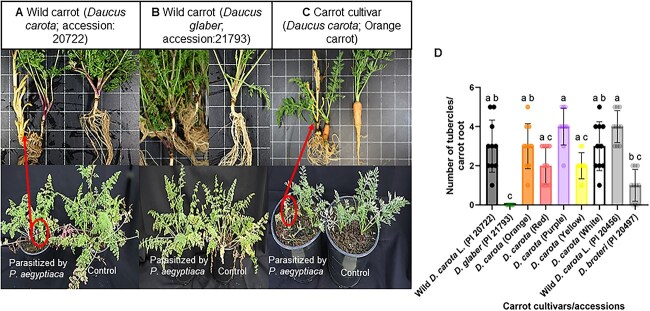
Differential rates of parasitism in wild and cultivated carrot. Photos from a greenhouse experiment with pots containing soil that was inoculated with *Phelipanche aegyptiaca* seeds or remained uninoculated (control) for three carrots: (A) wild carrot acc20722 (*Daucus carota* L.), (B) wild carrot acc21793 (*Daucus glaber*), and (C) cultivated orange carrot (*D. carota*). (D) Graph showing the number of *P. aegyptiaca* tubercles observed in a pot experiment with different cultivated (orange, red, purple, yellow, and white) carrots and the wild carrot accessions *D. glaber* (PI 21793), *D. carota* L. (PI 20456, PI 20722), and *Daucus broteri* (PI 20497). Data are shown as the mean ± SD. Each dot represents an individual pot (*n* = 10). Different letters indicate significant differences (Kruskal–Wallis test; *P* < 0.05), followed by Dunn’s multiple comparison test) in *P. aegyptiaca* growth across the various carrot cultivars and species.

A screening of wild carrot germplasm and carrot cultivars for response to parasitism by *P. aegyptiaca* was conducted at the Volcani Institute (ARO, Israel) resulting in the selection of a resistant accession of *Daucus glaber* (PI 21793). This accession showed complete resistance against the parasite under greenhouse conditions (Fig. 1). To examine potentially similar resistance mechanisms in other wild carrot species, we also investigated an accession (PI 341902) of *Daucus littoralis*, which is a close relative of *D. glaber* and exhibits similar growth patterns*.* A susceptible purple carrot cultivar (P0114; *D. carota*) was used as a control.

**Figure 2:**
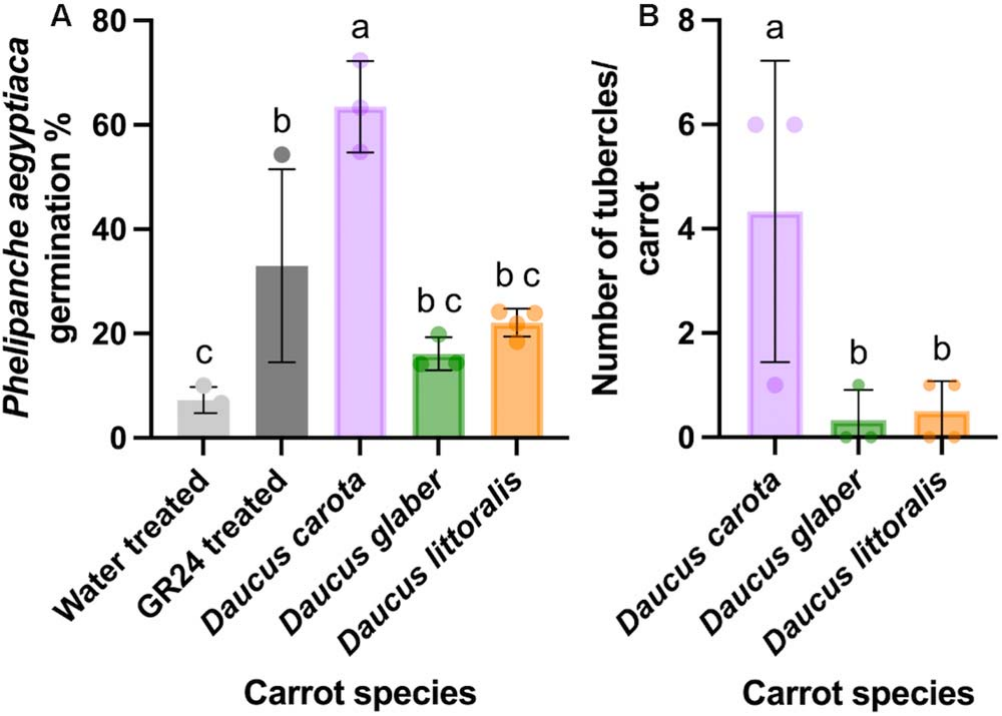
Impact of different carrot accessions on germination and tubercle development of *Phelipanche aegyptiaca*: (A) percent germination, and (B) tubercle development of *P. aegyptiaca* seeds in the presence of carrot accessions, *Daucus carota* (purple carrot; P0114), *Daucus glaber* (PI 21793), and *Daucus littoralis* (PI 341902). Control treatments consisted of *P. aegyptiaca* seeds without host plants and exposed to water only (negative control) or to GR24 (positive control). Data are shown as the mean ± SD. The dots represent independent replicates per treatment; *n* = 3 for all treatments except *n* = 4 for *D. littoralis*. Different letters indicate significant difference (one-way ANOVA; *P* < 0.05; Tukey’s HSD) in *P. aegyptiaca* growth across the various carrot species.

To determine the effect of the different carrot accessions on *P. aegyptiaca* seed germination and further development, carrot roots were inoculated with *P. aegyptiaca* seeds in a semi-hydroponic, polyethylene (PE) bag cultivation system (Goldwasser et al. 1997). Similar to the observations made at the Volcani Institute, *D. glaber* plants demonstrated resistance to *P. aegyptiaca* (Fig. 2). In particular, there was a reduction in both germination and tubercle development of *P. aegyptiaca* when grown with *D. glaber and D. littoralis* as compared to *D. carota.* Average germination rates of *P. aegyptiaca* in the presence of the wild carrot accessions (16% with *D. glaber* and 22% with *D. littoralis*) were above those of water-treated seeds in the negative control but about three- to four-fold lower than germination rates in the presence of *D. carota* roots (63%) (Fig. 2A). Seeds treated with the germination stimulant GR24 were used as a positive control. When comparing the number of tubercles that developed on the carrot roots, *D. carota* supported an average of about 9 to 12 times more tubercles per host plant than *D. littoralis* and *D. glaber*, respectively (Fig. 2B) where the numbers of tubercles ranged from only 1 to 0 per plant.

### Wild carrots appear to be deficient in stimulant production or exudation and are less susceptible to parasitism

**Figure 3:**
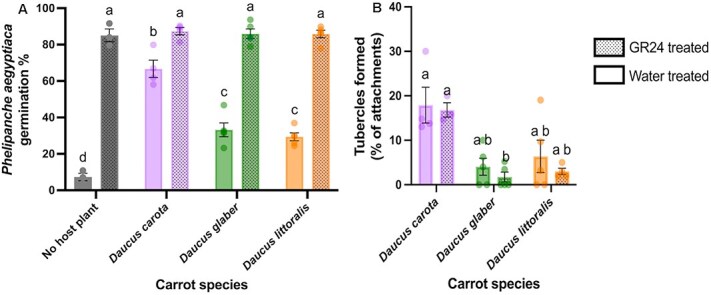
Impact of different carrot accessions on germination and tubercle development of *Phelipanche aegyptiaca* in the presence or absence of germination stimulant (GR24): (A) percent germination of *P. aegyptiaca*, and (B) rate of successful tubercle development from total *P. aegyptiaca* attachments, following pre-treatment with water or GR24 in the presence of different carrot accessions: *Daucus carota* (purple carrot; P0114), *Daucus glaber* (PI 21793), *Daucus littoralis* (PI 341902), and in a no-host control. Data are shown as the mean ± SD. Data points in (A) represent individual replicates per treatment (*n* = 3 for no-host controls, *n* = 4 for *D. carota*, and *n* = 5 for *D. glaber* and *D. littoralis* under both water, and GR24 pre-treatment conditions). Data points in (B) represent individual replicates per treatment (*n* = 4 for *D. carota*, *n* = 5 for *D. glaber* and *D. littoralis* under water pre-treatment and *n* = 3 for *D. carota*, *n* = 5 for *D. glaber*, and *n* = 4 for *D. littoralis* under GR24 pre-treatment). Different letters indicate significant difference (two-way ANOVA; *P* < 0.05; Tukey’s HSD) in *P. aegyptiaca* germination/growth across the various carrot species.

To further elucidate the role of germination stimulants in the resistance of wild carrots to *P. aegyptiaca*, two experiments were conducted. Both experiments used identical methods to determine parasite seed germination but differed in their subsequent assessment of tubercle formation. In both experiments, *P. aegyptiaca* seeds were pre-treated with water or the strigolactone analog GR24 and then exposed in PE bags to the roots of the different carrot species. In the presence of *D. carota*, average germination rates of *P. aegyptiaca* seeds were similar with or without GR24 treatment ranging from 64%–67% with water pre-treatment to 65%–87% upon exposure to GR24 (Fig. 3A and Supplementary Fig. S1A). In contrast, wild carrot roots had little stimulating effects on water-treated *P. aegyptiaca* seeds, whereas seeds pre-treated with GR24 displayed a significant increase in germination (Fig. 3A and Supplementary Fig. S1A). Germination rates in the two experiments ranged from 11%–33% with water-treated to 83%–85% with GR24-treated seeds in the presence of *D. glaber* and from 29% with water-treated to 78%–85% with GR24-treated seeds in the presence of *D. littoralis* (Fig. 3A and Supplementary Fig. S1A). In the absence of any host plant, *P. aegyptiaca* seeds treated with GR24 exhibited average germination rates of ~58%–85%, whereas water-treated seeds showed much lower germination, ranging from 2% to 7%. These similar findings in both experiments suggest that the resistance of wild carrots to *P. aegyptiaca* is largely caused by a deficiency in germination stimulant production or exudation.

The two experiments also assessed the extent of tubercle development following seed germination upon water treatment or stimulation by the addition of GR24. The first experiment (Supplementary Fig. S1B) recorded only the number of tubercles, without considering their proportion relative to total attachments. We found that *D. carota* supported the highest tubercle count in water-treated seeds, followed by GR24-treated seeds, with no significant differences between treatments (Supplementary Fig. S1B). Despite the GR24 treatment, average tubercle counts on *D. littoralis* and *D. glaber* roots remained ~1.5- to three-fold lower, respectively, than those on *D. carota* roots with GR24-treated seeds and three- to six-fold lower than counts on *D. carota* roots with water-treated seeds although these differences were not significant. As with *D. carota*, no substantial differences in the tubercle numbers were found between water- and GR24-treated seeds of each wild carrot accession.

The second experiment (Fig. 3) quantified both tubercles and total *P. aegyptiaca* attachments to determine the percentage of successful tubercle formation relative to the total attachments. This calculation provided a more informative measure of tubercle formation efficiency. Similar to the first experiment, *D. carota* showed the highest tubercle formation with both treatments. Interestingly, the percentage of successful tubercles formed from total attachments was slightly higher in water-treated seeds (Fig. 3B), indicating that increased germination did not directly enhance successful tubercle development. Tubercle formation rates on *D. littoralis* and *D. glaber* roots resulting from water-treated seeds were on average two- to five-fold lower than those on *D. carota*. Importantly, the percentage of tubercles observed on wild carrot roots exposed to GR24-stimulated seeds remained equally low with tubercle formation rates being significantly reduced on *D. glaber* roots (2%) compared to those on *D. carota* roots (17%) (Fig. 3B).

**Figure 4:**
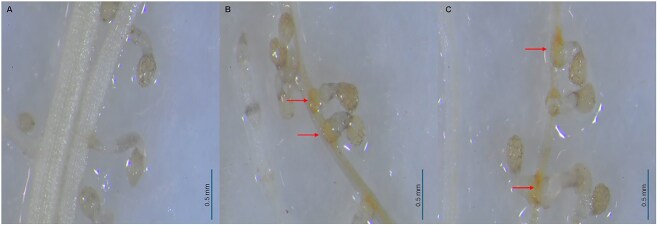
Differences in host response to *Phelipanche aegyptiaca* haustoria by different carrot accessions. Images show the site of attachment by *P. aegyptiaca* on (A) *Daucus carota*, (B) *Daucus glaber*, and (C) *Daucus littoralis*. Red arrows show induced responses in wild carrots at the point of attachment.

In summary, observations of *P. aegyptiaca* tubercle development in both experiments showed that tubercle formation efficiency was substantially reduced on wild carrot roots in comparison to cultivated carrots even when seed germination was pre-stimulated by GR24. This finding was further supported by the monitoring of visual phenotypes at the site of *P. aegyptiaca* seedling attachment. We observed that both wild carrot accessions exhibited a yellow-brown coloration at the attachment sites that was not seen in interactions with *D. carota* roots (Fig. 4).

### Effect of root exudates from different carrot accessions on germination of other root parasites

**Figure 5:**
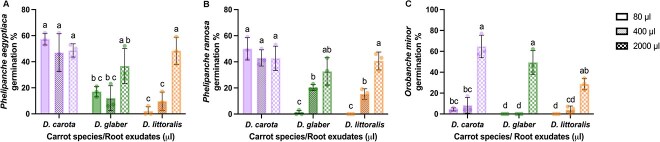
Germination rates of seeds of three parasitic plant species in the presence of root exudates from different carrot accessions. (A) *Phelipanche aegyptiaca*, (B) *Phelipanche ramosa*, and (C) *Orobanche minor* seed germination percentages were measured following exposure to varying volumes of root exudates (80, 400, and 2000 μl) from carrot accessions *Daucus carota* (purple carrot; P0114), *Daucus glaber* (PI 21793), and *Daucus littoralis* (PI 341902). Data are shown as the mean ± SD. The dots represent independent replicates per treatment; *n* = 3 for each carrot species and root exudate volume. Different letters indicate statistically significant differences in the interaction between carrot species and root exudate volumes (*P* < 0.05) based on two-way ANOVA followed by Tukey’s HSD for (A) *P. aegyptiaca* and (B) *P. ramosa;* and ANOVA on ranks (*P* < 0.05) followed by Sidak’s multiple comparison test for (C) *O. minor*. Germination responses to GR24 treatments across parasite species are provided in Supplementary Fig. S2.

We further tested *P. aegyptiaca* seed germination in the presence of root exudates of potted wild and cultivated carrot plants. The cultivation of carrots in pots allowed a convenient application of phosphorous deficient growth medium in order to increase the exudation of strigolactones. Seed germination was assessed on disks treated with extracts obtained from the root exudates (see Methods). In addition to *P. aegyptiaca,* we tested potential variations of germination of the two related parasite species, *P. ramosa* and *Orobanche minor* (Fig. 5). As with the germination tests in the presence of carrot roots in PE bags, there were significant differences in germination upon exposure to wild or cultivated carrot root exudates. The germination rates of *P. aegyptiaca* and *P. ramosa* were similar to each other and demonstrated high germination stimulation by *D. carota* exudates but less germination by exudates from *D. glaber* and *D. littoralis* depending on the amount of exudate*.* Application of increasing volumes of root exudate extracts (and presumably increasing amounts of germination stimulants in the assay) showed that even the lowest amount (80 μl) of *D. carota* exudate sample was sufficient to trigger significantly higher seed germination levels of both *Phelipanche* species than the same volume of exudate sample from wild carrot roots. The highest volume of exudate extract (2000 μl) was needed from wild carrots to elicit a similar response (Fig. 5A and B), indicating that germination stimulants occurred at low levels in these exudates. In contrast to the response of *P. aegyptiaca* and *P. ramosa*, the germination of *O. minor* was lower in response to all carrot extracts tested (Fig. 5C). It was necessary to use the 2000 μl volume of exudate extract to induce substantial germination of this species.

### Strigolactone profiles and quantitative differences between cultivated and wild carrots

**Figure 6:**
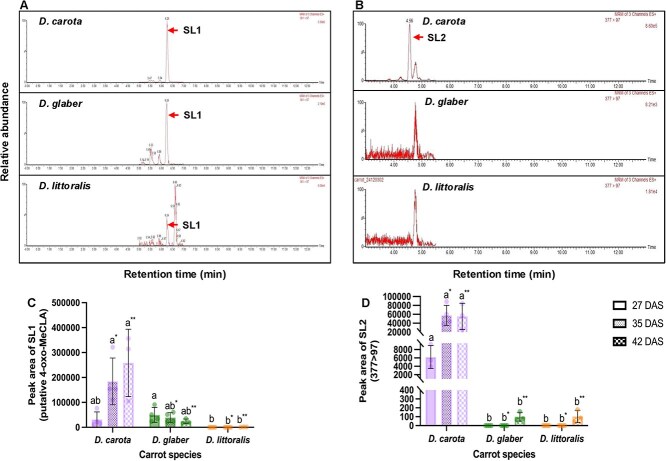
Detection and quantification of strigolactones in different carrot species at various time intervals (27, 35, and 42 DAS, days after sowing). (A) Chromatograms showing the detection of SL1 (putative 4-oxo-MeCLA) and (B) SL2 (*m*/*z* 377 → 97) extracted from the same root exudate volume of *Daucus carota* (purple carrot, P0114), *Daucus glaber* (PI 21793), and *Daucus littoralis* (PI 341902) at 35 DAS. (C) Bar graphs representing the peak areas of SL1 and (D) SL2 across carrot species at the respective time points. Data are shown as the mean ± SD. The dots represent independent replicates per treatment; *n* = 3–4 for each carrot species at 27 and 35 DAS, and *n* = 3 for each carrot species at 42 DAS. Different letters indicate statistically significant differences among carrot species within the same time point (*P* < 0.05). For SL1 at all-time points, and for SL2 at 27 and 35 DAS, significance was determined using the Kruskal–Wallis test followed by Dunn’s multiple comparison test. For SL2 at 42 DAS, one-way ANOVA followed by Tukey’s HSD was conducted. Letters without asterisks represent statistical differences at 27 DAS; letters with “*” denote differences at 35 DAS; and letters with “**” indicate differences at 42 DAS.

Two distinct carrot strigolactones, designated SL1 and SL2, were detected in extracts of root exudates from all carrot accessions examined in this study at various time intervals after sowing. For quantification, the primary multiple reaction monitoring (MRM) transitions used were *m*/*z* 361/97 for SL1 and *m*/*z* 377/97 for SL2, with peaks eluting at 6.2 and 4.5 min, respectively (Fig. 6A and B). Chromatograms for additional MRM transitions for SL1 (*m*/*z* 361/208 and *m*/*z* 361/247) and SL2 (*m*/*z* 377/179 and *m*/*z* 377/224), along with their product ion scans, are provided in the supplementary material (Supplementary Figs S3 and S4). Based on its mass fragmentation pattern, SL1 was putatively identified as 4-oxo-methyl-carlalactone (4-oxo-MeCLA) (Supplementary Fig. S5A) (Li et al. 2024).

SL1 was consistently present across all accessions, although its amounts varied significantly depending on the accession and the time interval (Fig. 6C). At 27 days after sowing (DAS), SL1 was detected in root exudates of *D. carota* and *D. glaber* and at very low levels in exudates of *D. littoralis*. At 35 and 42 DAS, average SL1 levels in exudates of *D. carota* exceeded those of *D. glaber* by five- to 10-fold, respectively, while they remained at trace levels in exudates of *D. littoralis*. SL2 was present at consistently high levels in exudates of *D. carota* but could not be detected in exudates of *D. glaber* and *D. littoralis* at 27 and 35 DAS and only at low levels at 42 DAS (Fig. 6D). Further analysis is required to confirm the identity of this strigolactone. In summary, we detected two strigolactones that accumulated at substantially higher levels in cultivated carrot root exudates than in exudates of wild carrots beyond one month of plant cultivation.

## Discussion

### Pre-attachment resistance in wild carrots against *P. aegyptiaca*

The present study reveals that wild carrot accessions of *D. glaber* and *D. littoralis* demonstrate resistance to *P. aegyptiaca* in comparison to the cultivated carrot species, *D. carota*. This resistance appears to be mediated by both pre- and post-attachment mechanisms, as evidenced by reduced seed germination rates and fewer successful tubercles (Figs 2 and 3 and Supplementary Fig. S1).

In principle, the reduced germination of *P. aegyptiaca* seeds may arise from factors such as differences in strigolactone composition (Yoneyama et al. 2015; Albanova et al. 2023; Li et al. 2023), lower strigolactone exudation (Dor et al. 2011, Pavan et al. 2016), or the presence of germination inhibitors in root exudates (Weerasuriya et al. 1993). Our data indicate that wild carrot species have reduced levels of strigolactones in their exudates. The difference in exudation can be influenced by various factors such as the age of the host plant and environmental biotic and abiotic factors (El-Halmouch et al. 2006, Ma et al. 2022). However, the germination assays in the PE bags in the current study were conducted without any biotic or abiotic modifications and both carrot cultivars and wild carrots were of the same age. One of the most important factors that control strigolactone production and exudation is nutrient availability to the host plant, especially phosphorus (Yoneyama et al. 2012). If there is less phosphorus availability, the plant exudes more strigolactones to recruit arbuscular mycorrhizal fungi (AMF) and increase phosphorus uptake (Clark and Bennett 2024). Therefore, the root exudates were collected from plants growing under nutrient deficiency to increase strigolactone exudation (K. Yoneyama et al. 2007a, K. Yoneyama et al. 2007b) that can lead to increased parasite germination (Jain and Foy 1992). Even under less nutrient availability, *P. aegyptiaca* and *P. ramosa* seed germination remained lower for root exudates from wild carrots compared to *D. carota* indicating reduced concentrations of germination stimulants. Thus, the volume of wild carrot root exudates had to be increased to obtain higher stimulant concentrations in the germination assay and achieve higher germination rates of both *Phelipanche* species (Fig. 6A and B). This finding is similar to observations in sorghum cultivars where elevated root exudate concentrations promoted *Striga* germination (Yoneyama et al. 2010), potentially because of increased strigolactone concentrations. Interestingly, *O. minor* exhibited lower germination rates even with the *D. carota* cultivar root exudates, suggesting a low abundance of favorable strigolactones for *O. minor* across all carrot species (Fig. 6C). Although *O. minor* can germinate in response to a broad spectrum of strigolactones (Huizinga and Bouwmeester 2023) and has been recognized as a parasite of carrot (Parker and Riches 1993), our study showed reduced sensitivity to all carrot-derived strigolactones.

With respect to the potential involvement of germination inhibitors, certain plants exude toxic compounds that are detrimental to germination and radicle development of root parasites (Jhu et al. 2023). For example, trioxazonane in the root exudates of fenugreek (*Trigonella foenum-graecum* L.) inhibited *O. crenata* germination (Evidente et al. 2007), and hydroxylated coumarins in sunflowers (*Helianthus annuus* L.) inhibited *Orobanche cernua* germination and induced browning of germinated seedlings (Serghini et al. 2001). However, when *P. aegyptiaca* seeds were pre-treated with GR24 to compensate for any lack of germination stimulant (similar to the method of Dor et al. 2011), wild carrots did not show any ability to reduce levels of *P. aegyptiaca* germination (Fig. 3 and Supplementary Fig. S1). This suggests that pre-attachment resistance is not attributable to toxic compounds but instead to reduced or altered strigolactone exudation (Dor et al. 2011).

### Non-canonical strigolactones and differential exudation in wild carrots

Two carrot strigolactones, termed SL1 and SL2, were detected in high abundance in the *D. carota* cultivar, whereas only trace amounts of these compounds were observed in wild carrot accessions. Interestingly, we identified SL1 as putative 4-oxo-MeCLA. This compound is considered a non-canonical strigolactone that does not belong to the strigol or orobanchol types, which are known to play a significant role in parasitic plant seed germination in other host plant species (Jia et al. 2018). 4-Oxo-MeCLA was identified recently in root exudates of rice and shown to play a role in root development and establishment of AMF symbiosis (Li et al. 2024). Our possible detection of this strigolactone in carrot suggests that it also occurs in dicot species.

Although germination of *Phelipanche* was reduced due to quantitative differences in SL1 and SL2 exudation, we assume that an altered composition of strigolactone profiles may also contribute to the differences in seed germination. Our preliminary search for alternative strigolactones may have detected additional compounds in exudates of the wild carrot accessions, but these findings require further verification. Analyses in other host plants such as maize have shown that differences in strigolactone profiles are linked to variations in susceptibility and resistance responses to the parasite (Yoneyama et al. 2015) and elicit varying responses from parasitic plants (Fernández-Aparicio et al. 2011, Bouwmeester et al. 2021). Alternative strigolactones in the exudates of wild carrots may help retain beneficial interactions with mycorrhizal fungi while having a limited role in stimulating parasite seed germination. Future studies should aim to fully elucidate the strigolactone profiles of wild carrots and investigate their effects on both parasitic and mutualistic interactions.

### Partial post-attachment resistance mechanism

In addition to pre-attachment resistance, wild carrots (*D. glaber* and *D. littoralis*) demonstrated partial post-attachment resistance, as shown by consistently fewer tubercles on these carrots than on *D. carota*, even when *P. aegyptiaca* germination was stimulated with GR24 (Figs 3 and 4). Similar findings have been reported in wild *Sorghum* accessions, where both pre-attachment and post-attachment resistance mechanisms were observed, with reduced *Striga* germination and haustorium initiation, as well as necrosis of *Striga* attachments on *Sorghum* roots (Mohamed et al. 2003, Rich et al. 2004, Ahmed et al. 2024). In carrot, only Zehhar et al. (2003) have reported post-attachment resistance in a Buror variety, specifically against *P. ramosa*, where necrosis of the parasite was linked to lignification of host cells.

In this study, a yellow-brown discoloration (indicative of a necrosis-like effect) was observed at the points of attachment in *D. glaber* and *D. littoralis* (Fig. 4). Similar discolorations have been documented in the semi-resistant Palaiseau carrot cultivar (Zehhar et al. 2003), and a reddish-brown coloration in the resistant vetch cultivar Popany (Goldwasser 2000). While this response does not completely prevent tubercle formation, it is associated with negative effects on the parasite's development and survival. The observed discoloration may indicate a hypersensitive response or the release of secretions by the parasite’s intrusive cells to overcome host barriers (Pérez-De-Luque et al. 2005, Albanova et al. 2023). These responses could either block further development of parasite attachments or interfere with host–parasite nutrient transfer, thereby restricting parasite growth (Pérez-De-Luque et al. 2005). However, further histochemical analysis is necessary to elucidate the exact mechanisms underlying the resistance observed in this study.

## Conclusion

This study emphasizes the importance of wild germplasm as a source of solutions against parasitic plants. Both pre-attachment and partial post-attachment resistance mechanisms were identified in wild carrots (*D. glaber* and *D. littoralis*) against root parasitic plants. Strigolactone analysis revealed significant quantitative differences, with cultivated carrots producing higher amounts of SL1 (putative 4-oxo-MeCLA) and SL2 than wild carrots, which substantially influenced parasite seed germination. Additionally, the presence of necrosis-like effects at parasite attachment sites in wild carrots indicates a partial post-attachment resistance mechanism that restricts tubercle formation and development. These findings enhance our understanding of the biochemical differences underlying host resistance to parasitic plants and highlight the value of wild carrot accessions as genetic resources for breeding or engineering parasitic plant-resistant cultivars. Some crosses between wild species and domesticated carrots produce viable seeds, although to date none have been successful between *D. glaber* or *D. littoralis* (2*n* = 20) and domesticated carrot (2*n* = 18, P. Simon, unpublished observations) as was observed in attempted crosses between carrot and *D. pusillus* (2*n* = 22; Camadro et al. 2008). In this case, identification of resistance mechanisms in the wild species provides essential information for editing carrot genomes for parasite resistance.

## Material and Methods

### Screening of wild and cultivated carrots to *P. aegyptiaca*

Carrot (*D. carota* subsp. *sativus*) cultivars of various colors, orange, red, purple, yellow, and white, and four wild accessions from three *Daucus* species (*D. carota* L.; *D. glaber* (Forssk.) Thell.; *D. broteri* Ten.) were obtained from the Israel Plant Gene Bank. Seeds were originally collected from different regions across Israel (Supplementary Table S1).

These carrot cultivars and accessions were grown under standard field irrigation and fertilization conditions in a greenhouse facility at the Newe Ya’ar Research Center in northern Israel. The plants were grown in 2 L pots containing potting mix (Newe Ya’ar soil, Chromic Haploxererts, fine clayey, montmorillonitic, thermic, 55% clay, 25% silt and 20% sand, 2% organic matter, and pH 7.2) that was either infested or non-infested with seeds of the parasitic weed *P. aegyptiaca.* The *P. aegyptiaca* seeds (50 mg/l) were mixed with the entire soil content. After ~12 weeks of growth, the carrot roots were excavated and cleared of soil to quantify the *P. aegyptiaca* tubercle attachments.

### Plant material for PE bag bioassays

To further understand the resistance mechanisms in resistant carrots, seeds of accession PI 21793 (*D. glaber*) were obtained from the Newe Ya’ar Research Center (Agricultural Research Organization), Ramat Yishay, Israel. Seeds of a wild carrot accession, PI 341902 (*D. littoralis*), and a purple carrot cultivar, P0114 (*D. carota*), were obtained from the seed collection at the USDA/ARS at Madison, WI. Plants were cultivated as described below. The carrot plants were inoculated with *P. aegyptiaca* seeds collected at the Newe Ya’ar Research Center (courtesy of Dr. Daniel Joel).

### *P. aegyptiaca* seed sterilization and conditioning

The seeds of *P. aegyptiaca* were surface sterilized through a sequential exposure to 70% ethanol for 30 s, 1% sodium hypochlorite solution for 20 min, and 0.01 M hydrochloric acid for 10 min, followed by three 10-min rinses in sterile double-distilled water. Subsequently, the seeds were placed on moistened glass fiber sheets (GFA, Whatman) sealed in a Petri dish and stored in dark conditions at 24°C for a week to undergo conditioning. For treatments involving the strigolactone analog GR24, conditioned seeds received a 1 ppm solution of racemic GR24 1 day prior to the inoculation on carrot roots. It is noteworthy that the GR24 treatment used as a positive control in the experiment shown in Fig. 2A was added to the nutrient solution in the bag rather than directly to conditioned seeds. This may have led to variable germination of *P. aegyptiaca* in that experiment and the treatment protocol was changed to that described above.

### PE bag bioassay

To inoculate carrot roots with *P. aegyptiaca* seeds, carrot plants were cultivated in two steps. Carrot plants were first planted in Sungro Professional growing mix and grown in pots for about 1 month under a short-day photoperiod (10 h) using Philips fluorescent bulbs (32 Watt-4100 Kelvin cool white), with temperature and relative humidity maintained at around 21°C and 53%, respectively. Germination of carrot seeds, especially from wild *Daucus* spp., was often poor and asynchronous, which limited the number of plants available for experiments. Plants of uniform age and size were then transplanted into the PE bag system (Goldwasser et al. 1997), which is used to allow observation of the stages of *Phelipanche* development. At this stage, the plants were transferred into a parasitic plant quarantine facility and exposed to a long-day photoperiod (15 h) using Philips fluorescent bulbs (32 Watt-4100 Kelvin cool white), maintaining a temperature of ~22°C. Plants were grown for a week in PE bags containing glass fiber sheets wetted with ¼ strength Hoagland’s solution (Hoagland and Arnon 1950) to adapt to the growth conditions and allow for new root growth. The Hoagland’s solution was supplemented as needed to maintain moisture. One week after transplanting, conditioned *P. aegyptiaca* seeds were inoculated close to the newly developed roots.

Conditioned *P. aegyptiaca* seeds usually start germinating 4–5 days after they perceive strigolactones. Therefore, the germination count was recorded a week after seed inoculation by counting the number of *P. aegyptiaca* seeds that germinated and calculating the percent of germinated seeds based on the total number of seeds in the bag. Attachment of the germinated seedlings to the carrot roots and tubercle formation of the parasite were observed 14 days after seed inoculation on the roots. Tubercle development was monitored for an additional week to assess further parasite growth. Initially, observations were focused on counting total *P. aegyptiaca* tubercles. Subsequently, for enhanced comparability across different PE bags and carrot species, the percentage of tubercles was calculated by dividing the total number of tubercles by the total number of *P. aegyptiaca* attachment events (including those that failed to develop after initial attachment).

### Collection of root exudates

Each carrot accession of *D. carota*, *D. glaber*, and *D. littoralis* was sown in vermiculite pots (volume, 700 ml; diameter, 12.4 cm; depth, 13.3 cm) and supplied with TT (Tadano and Tanaka) nutrients solution (K. Yoneyama et al. 2007b) twice over a 2-week period. Then, plants were grown under nutrient deficiency for 2 weeks to induce higher production/exudation of strigolactones. Tap water was poured onto the soil surface and 200 ml root exudates eluted from holes in the bottom of the pot were collected and extracted with ethyl acetate. For LC–MS/MS analysis, the ethyl acetate phase was dried over anhydrous MgSO_4_ and concentrated in vacuo. All crude samples were stored at 4°C until LC–MS/MS analysis.

### Germination assay using carrot root exudates

Germination assays with *O. minor*, *P. ramosa*, and *P. aegyptiaca* seeds were conducted in a manner similar to that reported previously (K. Yoneyama et al. 2007a). In short, treatment solutions were prepared as 80, 400, and 2000 μl aliquots from ethyl acetate extracts made from 100 ml root exudate. An acetone solution of the synthetic SL, GR24 (mixture of four stereoisomers, 10^−6^ M) was used as a positive control. The treatment solutions were added to a 5-cm Petri dish lined with a filter paper and the organic solvent was allowed to evaporate. Each treatment was initiated by placing in the dish a single glass-fiber disc (Whatman GF/A, Tokyo, Japan) carrying parasite seeds that had been previously conditioned by addition of sterile Milli-Q water and incubated at 23°C for 7 days. Discs were blotted to remove excess moisture prior to treatment. Finally, to each Petri dish 650 μl sterile water was added, dishes were sealed, enclosed in PE bags, and placed in the dark at 23°C for 5 days. Seeds were considered germinated when the radicle protruded through the seed coat.

### Strigolactone analysis by LC–MS/MS

For strigolactone analysis, root exudates were collected as previously described, with the exception that exudates were obtained from the same plants over multiple time points (27, 35, and 42 DAS). The collected exudates were analyzed via chromatographic separation on an ODS column (ACQUITY UPLC, BEH C18, 2.1 × 100 mm, 1.7 μm; Waters), using a water–methanol gradient containing 4% 50 mM ammonium acetate to promote ionization. Separation started at 35% methanol, followed by a 2 min gradient to 55% methanol, followed by a 13 min gradient to 95%, kept at 96% methanol for 2 min to wash the column and then back to 35% methanol for 3 min. The column was equilibrated at this solvent composition for 5 min before the next run. Total run time was 25 min. The column oven temperature was maintained at 40°C with a flow rate of 0.2 ml min^−1^ (sample injection volume of 1 μl).

For mass spectrometry analysis, the nebulizer and desolvation gas flows were 50 and 1200 l h^−1^, respectively. The capillary voltage was set at 0.5 kV, the source temperature at 120°C, and the desolvation temperature was 550°C. Fragmentation was performed by collision induced dissociation with argon at 3–5 × 10^−3^ mbar. The collision energy (CE) was optimized for each compound. MRM was used to detect each strigolactone. MRM transitions for the putative 4-oxo-MeCLA (SL1) eluting at 6.2 min were monitored for *m*/*z* 361/97 at a CE of 25 V, *m*/*z* 361/208 at a CE of 25 V, and *m*/*z* 361/247 at a CE of 15 V. For carrot SL2 eluting at 4.5 min, MRM transitions were monitored for *m*/*z* 377/97 at a CE of 25 V, *m*/*z* 377/179 at a CE of 20 V, and *m*/*z* 377/224 at a CE of 20 V with a cone voltage of 25 V. Data acquisition and analysis were performed using the MASSLYNX 4.1 software (Waters).

### Statistical analysis

All statistical analyses and graph creation were performed using GraphPad Prism version 10.2.3 except for non-parametric tests for two factorial experiments, for which R was used to conduct Analysis of variance (ANOVA) on ranks. For several experiments, we used one- or two-way ANOVA after confirming normality and equal variances. When assumptions were violated, we applied non-parametric tests such as Kruskal–Wallis or ANOVA on ranks.

For the initial study of carrot resistance to *P. aegyptiaca*, a Kruskal–Wallis test (*P* < 0.05), followed by Dunn’s multiple comparison test was used to compare the successful tubercle development across carrot accessions. The effects of different carrot accessions from various species on *P. aegyptiaca* germination and tubercle development were analyzed using a two-way ANOVA, with carrot accessions and synthetic strigolactone (GR24)/water treatment as fixed factors. Data were transformed as necessary to meet the assumptions of normality and equal variance. *Post hoc* comparisons were performed using Tukey’s honestly significant difference (HSD) test at a significance level of *α* = 0.05. Similarly, the root exudate assays for *P. aegyptiaca* and *P. ramosa* were analyzed using a two-way ANOVA, with exudates from different accessions and varying volumes as fixed factors, whereas ANOVA on ranks (*P* < 0.05) followed by Sidak’s multiple comparison test was used for the analysis of *O. minor* seed germination. Germination responses to GR24 treatments across parasite species for the root exudate assay also followed ANOVA on ranks followed by Sidak’s multiple comparison test. Differences in strigolactone levels between different carrot accessions were analyzed using one-way ANOVA or Kruskal–Wallis test followed by Dunn’s multiple comparison test. Since the focus was to evaluate differences among accessions rather than time points, the statistical analysis prioritized comparisons of strigolactone levels across carrot accessions at the same time point, and interaction effects over time were not calculated. For all experiments, details of replication numbers and analysis methods are included in the figure legends. Additional information on statistical analysis is provided in a Supplementary Meta Statistical Data file.

## Supplementary Material

pcp_2025_e_00082_File008_pcaf113

Supplementary_Meta_Statistical_Data

## Data Availability

All data supporting the findings of this study are included in the main text and supplementary materials.
