# Supplementary material for: Reduced strigolactone exudation as a key resistance mechanism in wild carrots against Phelipanche aegyptiaca
Source: Plant Cell Physiol. 2025 Sep 18;67(4):566–76. doi: 10.1093/pcp/pcaf113 (PMC13192447; doi:10.1093/pcp/pcaf113)
Supplement: pcp_2025_e_00082_File008_pcaf113 [file pcp_2025_e_00082_file008_pcaf113.pdf]

Supplementary material

Supplementary Table S1: Summary of plant material used in the screening of wild and cultivated carrots in Israel.

**Accession no.**	**Carrot species**	**Geographic region**	**Latitude (decimal)**	**Longitude (decimal)**
20456	*Daucus carota* L.	North Golan	33.116666	35.775277
20497	*Daucus broteri* Ten.	Lower Galilee	32.778055	35.414444
21793	*Daucus glaber* (Forssk.) Thell.	Akko Plain	32.905833	35.121944
20722	*Daucus carota* L.	Sharon Plain	32.311388	34.871111

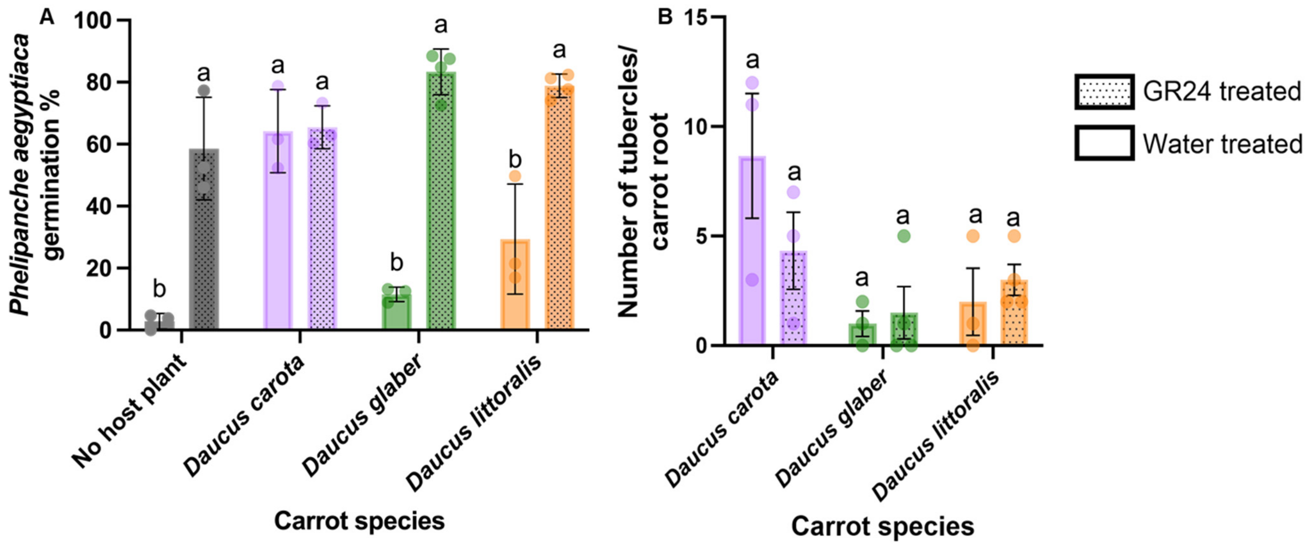

Figure S1: Impact of different carrot accessions on germination and tubercle development of *P. aegyptiaca* in the presence or absence of germination stimulant (GR24): (A) Percent germination, and (B) tubercle development of *P. aegyptiaca* seeds pre-treated with water or GR24 in the presence of carrot accessions, *D. carota* (Purple carrots; P0114), *D. glaber* (PI 21793), *D. littoralis* (PI 341902), and in the absence of a host plant. Data are shown as the mean ± SD. The dots represent independent replicates per treatment; n=3 for controls without a host plant, n=3 for *D. glaber*, *D. carota* and *D. littoralis* in the presence of *P. aegyptiaca* seeds pre-treated with water, and n=3 for *D. carota* and n=4 for *D. littoralis* and *D. glaber* in the presence of *P. aegyptiaca* seeds treated with GR24. Different letters indicate significant difference (two-way ANOVA; $p<0.05$; Tukey's HSD) in *P. aegyptiaca* growth across the various carrot species.

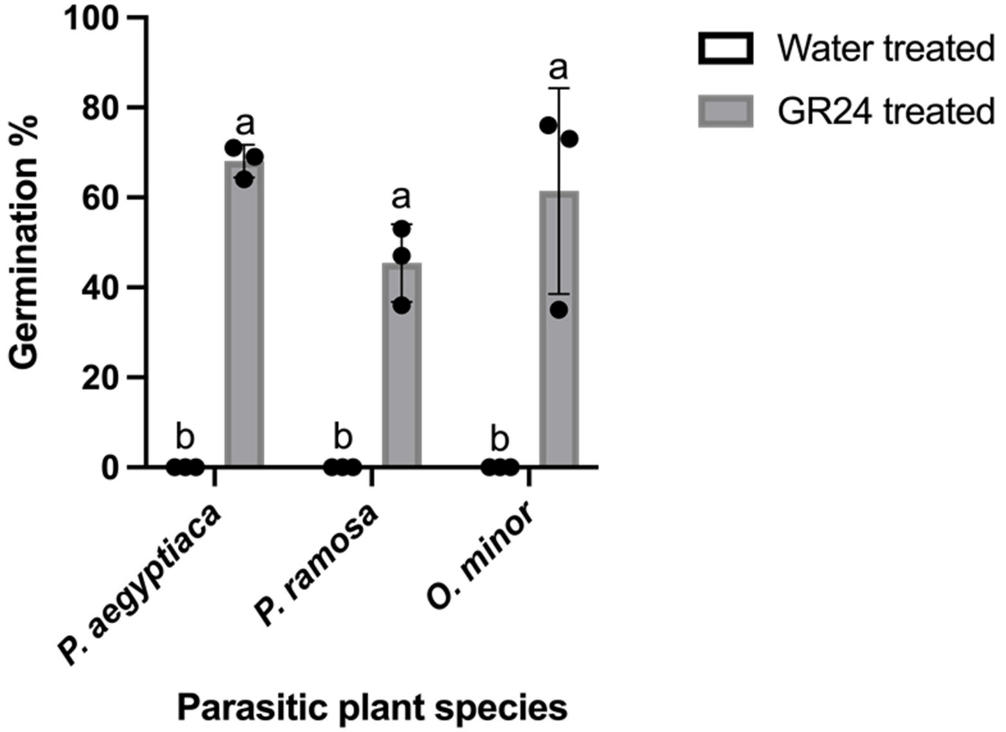

Figure S2: Germination response of three parasitic plant species to GR24 treatment in the carrot root exudate assay. Seeds of *P. aegyptiaca*, *P. ramosa*, and *O. minor* were treated with either water (negative control), 10^{-6} M GR24 (positive control; mixture of four stereoisomers in acetone), or carrot root exudates (Fig. 5). Bars represent mean germination percentage ± SD; individual dots indicate biological replicates (n=3). Statistical analysis was performed using ANOVA on ranked germination data, followed by pairwise comparisons using Sidak-adjusted *p*-values ($p<0.05$). Different letters indicate statistically significant differences between treatment groups.

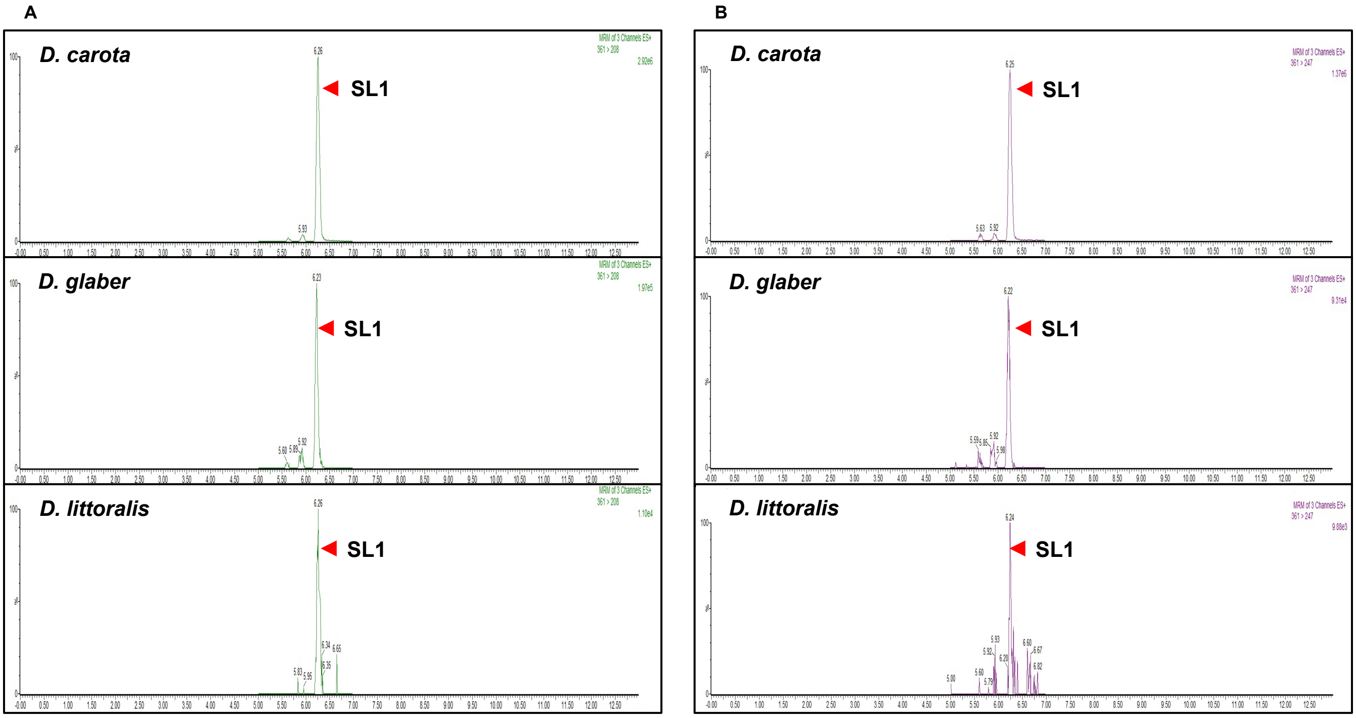

Figure S3: Multiple reaction monitoring (MRM) chromatograms for the detection of strigolactone SL1: (A) m/z 361 → 208, and (B) m/z 361 → 247 in root exudates of *D. carota*, *D. glaber*, and *D. littoralis*. The x-axis represents retention time (in minutes), and the y-axis shows relative abundance (normalized to a percentage of the SL1 peak).

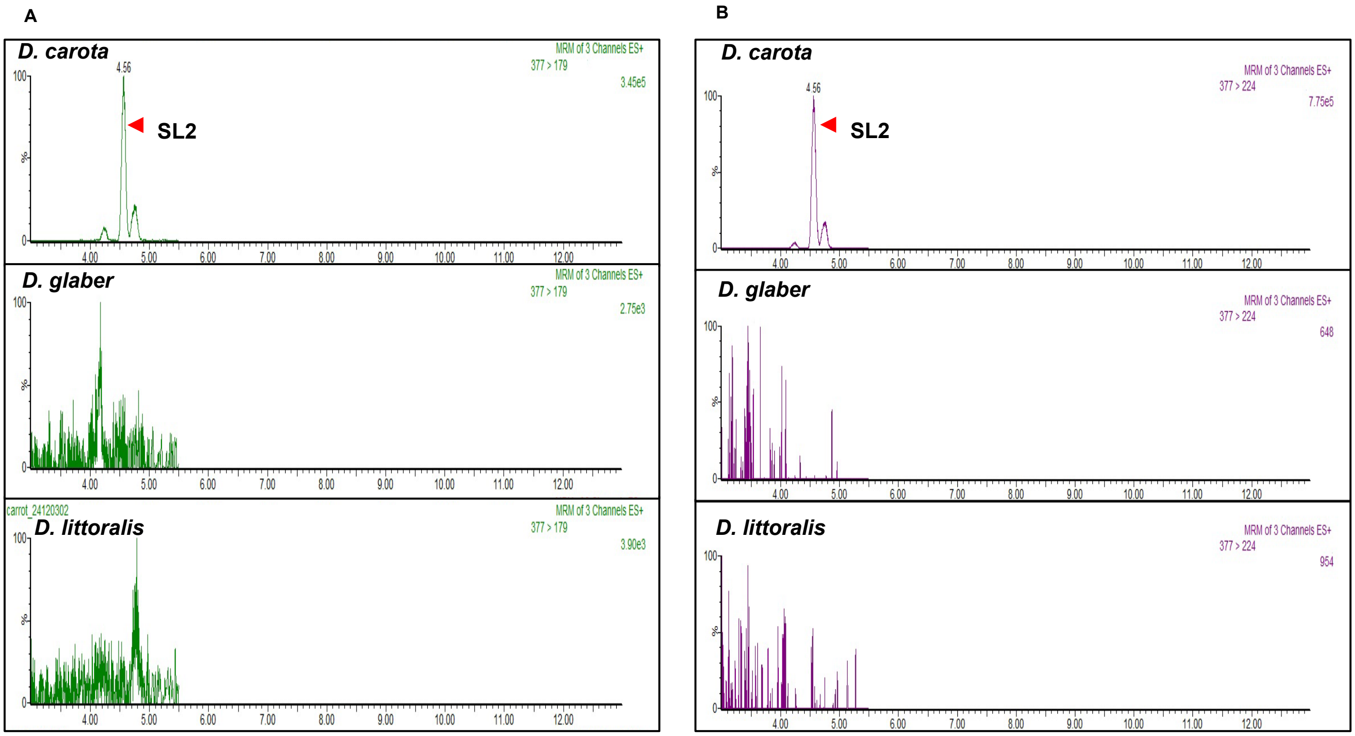

Figure S4: Multiple reaction monitoring (MRM) chromatograms for the detection of strigolactone SL2: (A) m/z 377 → 179, and (B) m/z 377 → 224 in root exudates of *D. carota*, *D. glaber*, and *D. littoralis*. The x-axis represents retention time (in minutes), and the y-axis shows relative abundance (normalized to a percentage of the SL2 peak).

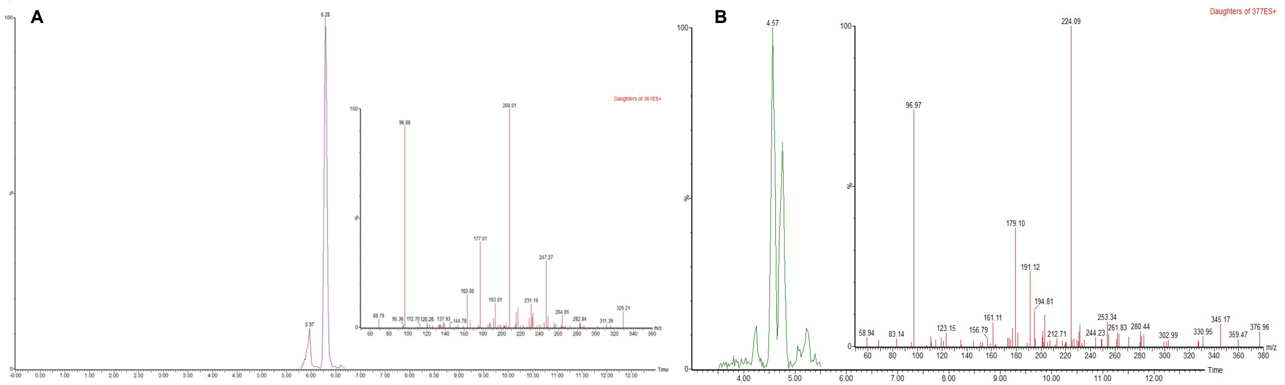

Figure S5: Product ion scans of two novel strigolactones identified in carrot root exudates. (A) Product ion scan of SL1 (putative 4-oxo-MeCLA) eluting at 6.2 minutes, showing the primary daughter ion at m/z 208 along with other characteristic fragments. (B) Product ion scan of SL2 eluting at 4.5 minutes, highlighting the major daughter ion at m/z 224 and associated fragment ions.
